# Testing strategies for genomic selection in aquaculture breeding programs

**DOI:** 10.1186/1297-9686-41-37

**Published:** 2009-06-30

**Authors:** Anna K Sonesson, Theo HE Meuwissen

**Affiliations:** 1 Nofima Marine AS, P.O. Box 5010, 1432 Ås, Norway; 2 University of Life Sciences, P.O. Box 5003, 1432 Ås, Norway

## Abstract

**Background:**

Genomic selection is a selection method where effects of dense genetic markers are first estimated in a test population and later used to predict breeding values of selection candidates. The aim of this paper was to investigate genetic gains, inbreeding and the accuracy of selection in a general genomic selection scheme for aquaculture, where the test population consists of sibs of the candidates.

**Methods:**

The selection scheme started after simulating 4000 generations in a Fisher-Wright population with a size of 1000 to create a founder population. The basic scheme had 3000 selection candidates, 3000 tested sibs of the candidates, 100 full-sib families, a trait heritability of 0.4 and a marker density of 0.5N_e_/M. Variants of this scheme were also analysed.

**Results:**

The accuracy of selection in generation 5 was 0.823 for the basic scheme when the sib-testing was performed every generation. The accuracy was hardly reduced by selection, probably because the increased frequency of favourable alleles compensated for the Bulmer effect. When sib-testing was performed only in the first generation, in order to reduce costs, accuracy of selection in generation 5 dropped to 0.304, the main reduction occurring in the first generation. The genetic level in generation 5 was 6.35σ_a _when sib-testing was performed every generation, which was 72%, 12% and 9% higher than when sib-testing was performed only in the first generation, only in the first three generations or every second generation, respectively. A marker density above 0.5N_e_/M hardly increased accuracy of selection further. For the basic scheme, rates of inbreeding were reduced by 81% in these schemes compared to traditional selection schemes, due to within-family selection. Increasing the number of sibs to 6000 hardly affected the accuracy of selection, and increasing the number of candidates to 6000 increased genetic gain by 10%, mainly because of increased selection intensity.

**Conclusion:**

Various strategies were evaluated to reduce the amount of sib-testing and genotyping, but all resulted in loss of selection accuracy and thus of genetic gain. Rates of inbreeding were reduced by 81% in genomic selection schemes compared to traditional selection schemes for the parameters of the basic scheme, due to within-family selection.

## Background

In current family-based aquaculture breeding schemes, many important traits are tested on the sibs of the candidates. The test information is used to calculate breeding values for the selection of parents [1]. Only 50% of the genetic variation *i.e. *the between family variation, is used in these schemes, which are employed for traits that cannot be measured on the selection candidates such as disease challenge testing and slaughter quality traits.

Dense marker maps and high-throughput genotyping have become increasingly available in aquaculture species. Genomic selection is a selection method where the effects of dense genetic markers are first estimated in a test population and later used to predict breeding values of selection candidates [2]. The sib-test design can be used for marker-assisted selection [3] or genomic selection, where the association between markers and phenotypes is estimated in the sibs of the candidates, and the candidates are selected on breeding values that result from summing the estimates of the effects of their marker alleles. For aquaculture breeding schemes, genomic selection may prove very useful, because the breeding goals include many traits that are based on information from the sibs and not from the candidates, and therefore genomic selection can result in increased accuracy of selection for those traits by using both between- and within-family genetic variances.

The aim of this paper was to investigate genetic gains, inbreeding and the accuracy of selection in a general genomic selection scheme for aquaculture, where selection is based on information from the sibs of the candidates. Schemes with different numbers of candidates and test animals (sibs of the candidates) and with different heritabilities of the trait under selection were compared by computer simulation. In addition, the importance of performing the sib-test every generation, which is costly for the breeding program, was assessed. Finally, the effect of selection on the accuracy of selection was evaluated.

## Methods

### Population

A population with an effective population size (N_e_) of 1000 was simulated for 4000 generations according to the Fisher-Wright population model [4,5]. Five hundred males and 500 females were randomly selected and mated using sampling with replacement.

Among the individuals of the last of these 4000 generations, 100 males and 100 females were randomly selected to create 100 full-sib families, which each produced 30 or 60 progeny to form generation Gen0. These progeny were selection candidates and were not performance tested. However, in addition to these selection candidates, every family also produced 30 or 60 full-sibs, which entered into a sib-test where they were performance tested. One hundred sires and 100 dams were selected from the candidates to produce generation Gen1 by either (a) random selection, whereby a sire and a dam were randomly sampled with replacement (RAND) or (b) directional selection, whereby sires and dams with the highest genome-wide breeding values (see Calculation of phenotypic values and true and estimated breeding values) were selected without any restriction on the number of parents selected from each family. Again each of the 100 sires was mated to one of the 100 dams, using sampling without replacement, to produce 30 or 60 full-sib selection candidates and 30 or 60 sib-test progeny in generation Gen1. This scheme was repeated until generation Gen10. Hence, with the number of families, Nfamilies, being 100, the total number of candidates, Ncand, was 3000 or 6000 and the total number of sib-test progeny, Ntested, was 3000 or 6000. In one scheme, Nfamilies = 200, Ncand = 3000 and Ntested = 3000.

### Reduction of the number of sib-tests

The idea here was to reduce the number of sib-tests by not performing a sib-test every generation. Four different strategies of sib-testing were compared:

EVERY GENERATION (EVERY-GEN): sib-testing was performed in every generation Gen0-Gen10 as described above in 2.1.

FIRST GENERATION (FIRST-GEN): sib-testing was performed in generation Gen0.

EVERY SECOND GENERATION (EVERY-2GEN): sib-testing was performed in the odd generation numbers Gen1, Gen3, .., Gen9.

FIRST 3 GENERATIONS (FIRST-3GEN): sib-testing was performed only during the first three generations Gen0-Gen2.

### Genome

Individuals had a diploid genome with ten 100 cM chromosomes. Recombinations were sampled at random positions on the chromosome assuming the Haldane mapping function. All polymorphisms were generated during the 4000 generations of the Fisher-Wright population model, where a mutation rate of 10^-9 ^per nucleotide was assumed and the number of nucleotides per cM was 1000000. This effectively resulted in the infinite sites mutation model [6], *i.e. *every mutation occurred at a unique position and created a bi-allelic SNP. This mutation process generated numerous SNP, among which 100 per chromosome were sampled randomly as a QTL (sampling without replacement from the SNP with minor allele frequency (MAF) >0.05), and among the remaining SNP, the 1000 with the highest MAF were chosen as genetic markers. The latter resulted in a total of markers of Nmarkers = 10000 spread over 1000 cM. Reduced numbers of markers were obtained by taking every 10^th ^marker and every 2^nd ^marker, resulting in a total of Nmarkers = 1000 and 5000 markers, respectively.

The allelic effects of the QTL alleles were sampled from the gamma distribution with a shape parameter of 0.4 and a scale parameter of 1.66 [7]. The QTL effects were assumed to be either positive or negative with a probability of 0.5, because the gamma distribution only gives positive values. After sampling, these QTL allelic effects were standardised so that the total genetic variance was 1.0 in Gen0.

The expected average linkage disequilibrium (*R*^2^) between two adjacent markers can be approximated by Hill [8]:



where *c *is the distance between adjacent loci, here on average 0.001 Morgan, which resulted in R^2 ^= 0.342. In generation Gen0, the realised average *R*^2 ^between adjacent markers was 0.374, which is slightly higher than Hill's approximation, probably due to the selection of the markers on their MAF.

### Calculation of phenotypic values and true and estimated breeding values

The true breeding value of an individual was calculated as:



where x_ijk _is the number of copies that individual i has at the j^th ^QTL position and k^th ^QTL allele, and g_jk _is the effect of the k^th ^QTL allele at the j^th ^position. The phenotypic values of the individuals in the sib-test were simulated by adding an error term sampled from a normal distribution to the true breeding value (*TBV*_*i*_):



where ε_*i *_is an error term for animal i, which was normally distributed (0, σ^2^_e_) and σ^2^_e _was adjusted so the heritability was 0.1 or 0.4.

Marker effects were predicted using the BLUP method described in [2]. The statistical model used to estimate the marker effects was:



where y_i _is the record of test individual i; μ is the overall mean; X_ij _denotes the marker genotype: 0 denotes that the individual is homozygous for the first allele; 1/√H_j _denotes that it is heterozygous; and 2/√H_j _denotes that it is homozygous for the second allele, where H_j _is the marker heterozygosity and thus dividing by √H_j _standardises the variance of the X_ij _to 1; a_j _is the random effect of the j^th ^marker and Var(a_j_) is assumed 1/Nmarkers (total genetic variance was standardised to 1.0); e_i _is a random residual.

Genome-wide breeding values were estimated by summing the effects of the markers:



The accuracy of selection (acc) was calculated as the correlation between true and estimated breeding values. The acc was calculated for all schemes, also for the RAND scheme, although the *EBV*_*i *_were not used for selection in RAND.

### Statistics

Selection schemes were run for ten generations (Gen1-Gen10) and summary statistics for each of the schemes are based on 50 replicated simulations. The breeding schemes were compared for the genetic level (G, expressed in genetic standard deviation units of generation Gen0 (σ_a_)), genetic gain (ΔG), accuracy of selection (acc), genetic variance, and level and rate of inbreeding (ΔF). Inbreeding was calculated based on pedigree, assuming that the Gen0 individuals are unrelated base parents. The values of these variables were either shown in figures over generations Gen1-Gen10 or in tables with the values in generation 5, when the Bulmer effect had stabilised, and inbreeding had started to build up in the population.

## Results

### Basic scheme

**Figure 1:**
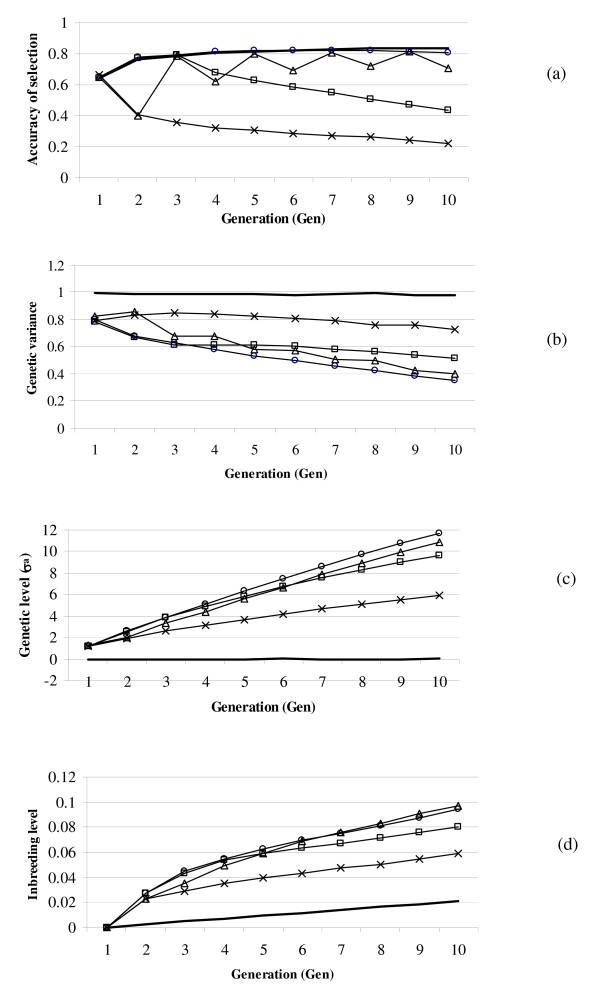
**Accuracy of selection, genetic variance, genetic level and inbreeding level for the basic scheme**. Accuracy of selection (a), genetic variance (b), genetic level (c) and inbreeding level (d) for schemes when sib-testing was every year (EVERY-GEN; circles), every second year (EVERY-2GEN; triangles), only the first year (FIRST-GEN; cross) or the first three years (FIRST-3GEN; squares); Random selection (solid line); Ncand = 3000, h^2 ^= 0.4, Nfamilies = 100, Ntested = 3000.

Accuracy of selection was the highest for EVERY-GEN and increased from 0.647 to approximately 0.820 over generations (Figure 1a), due to the increased amount of information on marker effects that becomes available. When phenotypic and genotypic testing was only in the first generation, as for FIRST-GEN, accuracy of selection decreased rapidly over generations and was only 0.304 in generation Gen5. This reduction in accuracy of selection is mainly because of changes in the linkage disequilibrium between marker and QTL. Especially, spurious LD [9], which is not due to linkage, changes quickly over generations. When the sib-test was performed every second generation, as for EVERY-2GEN, the accuracy of selection fluctuated, being almost as high as for EVERY-GEN in the generations with sib-testing and lower in the other generations. However, fluctuations decreased over generations, probably because the selection pressure increased favourable marker alleles, and then, their effects became increasingly accurately estimated. For FIRST-3GEN, the accuracy of selection was as high as for EVERY-GEN until generation 3, as expected, and thereafter, the accuracy of selection decreased. However, the reduction during the first generation after selection had stopped was not as large as for EVERY-2GEN, probably because more generations of information on marker effects had built up. In general, the reduction in accuracy of selection after sib-testing had stopped was larger when there were fewer generations of information *i.e. *the reduction in accuracy of selection was the largest for FIRST-GEN, thereafter EVERY-2GEN, and FIRST-3GEN.

The RAND scheme had an accuracy of selection very similar to EVERY-GEN.

The genetic variance was reduced over generations of selection, except for the RAND schemes, as expected (Figure 1b). The genetic variance decreased most for the EVERY-GEN scheme, with the highest accuracy of selection, and least for the FIRST-GEN scheme. For FIRST-GEN, the largest reduction was between generations Gen1 and Gen2.

The accuracy of selection and genetic variance resulted in the highest genetic level for EVERY-GEN and the lowest for FIRST-GEN, as expected (Figure 1c). In generation Gen5, the genetic level was 6.35σ_a _for EVERY-GEN and 3.69 σ_a _for FIRST-GEN. For FIRST-3GEN, the genetic level was 5.82 σ_a_. However, the genetic level of FIRST-3GEN became over time increasingly lower than that of EVERY-GEN. The genetic level of EVERY-2GEN in generation Gen5 was 5.66 σ_a _and the difference in genetic level between EVERY-GEN and EVERY-2GEN increased over generations.

Inbreeding levels were also the highest for EVERY-GEN, probably because it had the highest accuracy of selection (Figure 1d). For FIRST-3GEN, the inbreeding level was as high as for EVERY-GEN until generation Gen4, and decreased thereafter, also because of lower accuracy of selection in the generations after the sib-testing had stopped.

### Effect of marker density

**Figure 2:**
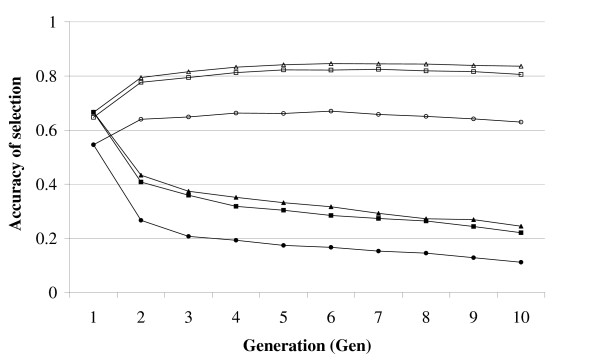
**Accuracy of selection with different numbers of markers and sib-testing strategies**. Accuracy of selection for schemes with 1000 (circles), 5000 (squares) or 10000 (triangles) markers when sib-testing was every year (EVERY-GEN; open) or only the first year (FIRST-GEN; filled); Ncand = 3000, h^2 ^= 0.4, Nfamilies = 100, Ntested = 3000.

The effect of different marker densities on the accuracy of selection was large *i.e. *when increasing Nmarkers from 1000 to 5000, the accuracy of selection increased from 0.661 to 0.823 using EVERY-GEN (Figure 2). However, when increasing Nmarkers from 5000 to 10000, the accuracy of selection hardly increased and was 0.842 in Gen5 with Nmarkers = 10000. For FIRST-GEN, the accuracy of selection decreased relatively much faster for the lowest number of markers *i.e. *1000 (-51%) than for 5000 (-39%) or 10000 (-35%) markers. This suggests that if the markers are sufficiently close to the QTL, the linkage disequilibrium changes less and thus the estimates of the marker effects remain more accurate.

### Effect of heritability and numbers of candidates, tested sibs and families

Table 1 shows the sensitivity of the results to lower heritability of the trait, higher number of candidates and tested sibs, and higher number of families. Decreasing the heritability from h^2 ^= 0.4 to 0.1, decreased ΔG_5 _by 7%. Similarly, acc_5 _was 10% lower for the scheme with low heritability, but ΔF_5 _was 50% higher for the scheme with low heritability, because of the increased between-family selection. The vg_5 _was 5% higher for the scheme with a low heritability, because of lower selection accuracy.

**Table 1:** Effect of heritability and numbers of candidates, tested sibs and families

	ΔG_5 _(se)	acc_5 _(se)	ΔF_5 _(se)	vg_5 _(se)
Basic scheme^1^	1.22 (0.009)	0.823 (0.003)	0.008 (0.002)	0.532 (0.006)
Lower heritability, h^2 ^= 0.1		0.742 (0.004)	0.012 (0.002)	0.561 (0.009)
Higher number of candidates, Ncand = 6000	1.34 (0.019)	1.13 (0.012)	0.009 (0.002)	0.468 (0.010)
Higher number of tested sibs, Ntested = 6000	1.24 (0.009)	0.845 (0.002)	0.007 (0.002)	0.540 (0.007)
Higher number of families, Nfamilies = 200	0.99 (0.010)	0.749 (0.004)	0.004 (0.002)	0.642 (0.007)

Genetic gain (ΔG_5_), accuracy (acc_5_), rate of inbreeding (ΔF_5_) and genetic variance (vg_5_) in generation 5 for schemes with different numbers of candidates (Ncand = 3000 or 6000), tested sibs (Ntested = 3000 or 6000) and families (Nfamilies = 100 or 200) and heritability levels (0.1 or 0.4). EVERY-GEN testing strategy was used.

^1^h^2 ^= 0.4, Ncand = 3000, Ntested = 3000, Nfamilies = 100

Increasing the number of selection candidates from Ncand = 3000 to 6000 increased ΔG_5 _by 10%. The increased genetic gain was mainly the result of higher selection intensity. However, this also resulted in larger increases of allele frequencies of favorable alleles, which in turn resulted in more accurate estimates of these alleles. Thus acc_5 _was somewhat higher *i.e. *0.837 for the scheme with 6000 selection candidates compared to 0.823 for the scheme with 3000 selection candidates. Increasing the number of tested sibs from Ntested = 3000 to 6000 increased acc_5 _by only 2.7%, which hardly affected neither ΔG_5_, vg_5 _nor ΔF_5. _This suggests that Ntested = 3000 is sufficient when h^2 ^= 0.4.

Increasing the number of families from Nfamilies = 100 to 200, reduced ΔF_5 _from 0.008 to 0.004, because of the increased numbers of sires and dams selected. The latter resulted in higher genetic variance, which was 21% higher for the scheme with Nfamilies = 200 than with Nfamilies = 100. The acc_5 _was 9% and ΔG_5 _19% lower for the scheme with Nfamilies = 200 than with Nfamilies = 100, because selection intensity was reduced.

## Discussion

This simulation study examines the accuracies of selection and genetic gain that can be attained with genomic selection sib-testing schemes in aquaculture. Various strategies were evaluated to reduce the amount of sib-testing, but all resulted in a loss of accuracy of selection and thus of genetic gain. Whether these reductions in genetic gains are acceptable will depend on the relative sizes of the reduced benefits and the savings due to less sib-testing. In schemes where selection is for an index combining growth and sib-testing traits, the reduction in accuracy of selection and thus genetic gain will be less than that which was found here due to the reduced importance of the sib-testing traits. How much the accuracy of such an index will be reduced can be assessed using the results in Table 1 and selection index calculations. The general picture that emerges from Figure 1a is, however, that continuous phenotypic and genotypic testing is important to maintain the accuracy of the genome-wide breeding values over generations. The results showed that the number of previous generations of sib-testing affected the level of accuracy proportionally, such that the reduction in accuracy of selection over generations was the largest for FIRST-GEN, thereafter EVERY-2GEN, FIRST-3GEN and finally EVERY-GEN. It may be noted that the reduction in accuracy of selection was substantially larger for the first generation after sib-testing had stopped compared to later generations. This may be because within a generation, markers merely explaining family effects can be used for the prediction of breeding values, whereas across generations, the family effects decay. These results agreed with those of [10]. (Solberg, T.R., Sonesson, A.K., J. A. Woolliams, Ødegård, J., Meuwissen, T.H.E: Persistence of estimates of genome-wide markers over generations when including a polygenic effect, submitted) found a much smaller reduction in the accuracy over generations than we saw here. However, their study did not include the effect of selection. Also, the BayesB method of [2] was used instead of BLUP, which may have increased the weight given to markers that are in close LD with the QTL. Habier *et al. *[11] found that the accuracy of selection did not decrease as much with BayesB as with BLUP, because BayesB gives more weight to the LD when estimating genome-wide breeding values. Hence, the reduction observed in our study may be smaller if the BayesB method was used.

During later generations (say generations Gen6-10), the accuracy of selection was based on LD and its reduction was much smaller, indicating that the LD decayed slowly. Still, the low value of the selection accuracy suggests that in genomic selection breeding schemes also, the prediction of family effects is important and that breeding designs where family effects can be accurately predicted are important. This implies that the relationship between the individuals in the test group and the selection candidates should be as high as possible.

When decreasing heritability from 0.4 to 0.1, acc_5 _decreased from 0.823 to 0.742 for the basic scheme with 30 sibs per family, *i.e. *a reduction of 0.08. The expected accuracy of selection for a traditional BLUP scheme is 0.66 for a heritability of 0.4 and 0.55 for a heritability of 0.1, *i.e. *a reduction of 0.11[12]. Hence, the effect of heritability on the accuracy of selection is somewhat larger for traditional BLUP schemes than for a scheme with genome-wide breeding values, which is in agreement with the literature on genomic selection [13] and marker assisted selection ([14] and others). The results also showed that the accuracy of selection increased by 25 and 35% when using genomic selection compared to traditional BLUP for the scheme with a heritability of 0.4 and 0.1, respectively. See [13] for a more detailed comparison of traditional and genomic selection schemes.

Truncation selection for traditional BLUP breeding values [15] resulted in a ΔF of 0.043 for the parameters of the basic scheme. Thus, ΔF was dramatically reduced by 81% for the genomic selection schemes compared to the traditional BLUP breeding schemes, although in practical breeding schemes, a way of reducing rates of inbreeding would probably be used, *e.g. *reducing the use of number of parents from single families or using optimum contribution selection. The reduced ΔF for genome-wide breeding values was probably due to the increased possibilities for within-family selection in genomic selection schemes, whilst this is not possible in traditional sib-testing schemes. It was also due to a stronger Bulmer effect since a more accurate selection increases the Bulmer effect, which leads to less between-family variance and thus more within-family selection [16].

In this study, we used 1000, 5000 and 10000 markers on a genome size of 1000 cM and an effective population size (N_e_) of 1000. Hence, the marker density was 0.1, 0.5 and 1N_e_/M, which is not very high, but probably in accordance with what a first-generation SNP chip would include for most aquaculture species. For example, with a total genome size of 30M for salmon [17] and an assumed N_e _of 1000 (see discussion [18]), 1N_e_/M implies a SNP chip of 30000 markers. Furthermore, Figure 2 shows that increasing the marker density from 0.5 to 1N_e_/M produces little extra gain. The low number of records relative to the N_e _explains this plateau and such a plateau has also been reported by [19] with a SNP marker density of around 4N_e_/M.

The effect of selection on its accuracy was rather small here (EVERY-GEN versus RAND). This result is contrary to that of Muir [10], but he did not update the marker effects every generation, which made the accuracy decay rapidly. In the EVERY-GEN scheme, marker effects were updated every generation, but still one might expect that the Bulmer effect, which reduces the genetic variance (Figure 1b), would also reduce the accuracy of selection, because the signal to noise ratio is reduced. However, a selective genotyping effect occurs, due to selection, resulting in increased frequency and higher accuracy of marker alleles with positive effects. The Bulmer and selective genotyping effects seem to balance each other out approximately, resulting in an accuracy that is hardly reduced by selection.

For practical aquaculture breeding schemes, the results of this study have the following implications, which could lead to a new design of the breeding programs. (i) Genotyping with genomic selection generates extra costs, which can be partly recovered by higher ΔG due to the increased accuracy by genomic selection compared with conventional BLUP breeding values. Here, we found an accuracy of around 0.82 for genome-wide breeding values compared with 0.66 for conventional BLUP breeding values, for a sib-test with 30 sibs tested per family and a heritability of 0.4. (ii) The breeding companies could reduce phenotyping costs if they cancelled all sib-tests of the candidates in one generation, like in EVERY-2GEN. In many salmon breeding schemes, most traits are actually measured on the sibs of the candidates, and phenotypic testing for all these disease and slaughter tests constitute a large part of the cost of the breeding program. However, the reduction of the sib-testing reduces ΔG_5 _by 16% from 3.80 σ_a _for EVERY-GEN to 3.19 σ_a _for EVERY-2GEN. FIRST-GEN reduces ΔG_5 _even more. An alternative is to use field data, *e.g. *slaughter house data or practical disease outbreaks, to estimate the association between markers and phenotypes. The data must come from the population under selection and represent animals closely related to the selection candidates, because Figure 1 shows that since the relationship between test individuals and individuals whose EBV are estimated decreases, the accuracy of selection decreases. (iii) Genotyping all selection candidates, *e.g. *100 families times 30 candidates per family = 3000 individuals, is probably not feasible in practice. Instead, pre-selection on *e.g. *growth and maybe other traits could be a way to reduce the number of candidates to be genotyped. However, this pre-selection step needs to be optimised, in order to get the desired weight on the traits in the pre-selection step relative to the other traits. Also the sib-tested individuals need to be genotyped in the breeding scheme presented. Here the number of genotypes could probably be reduced by a factor of 5 without a substantial loss of accuracy of selection using a selective genotyping strategy [20].

The results show that the accuracy of selection is mainly affected by the following parameters of the breeding scheme:

1. In general, accuracies are highly affected by the number of generations with information on marker effects. For the basic scheme, with 3000 selection candidates, 3000 tested sibs of the candidates, 100 full-sib families and a trait heritability of 0.4, accuracy of selection increased from 0.647 to approximately 0.823 over generations for EVERY-GEN. When sib-testing was only in generation 1, as in the FIRST-GEN in order to reduce costs, accuracy of selection dropped rapidly and was only 0.304 in generation 5 for the basic scheme.

2. Genomic selection, using EVERY-GEN, showed a higher accuracy of selection than the theoretical maximum of 0.71 of a conventional sib-testing scheme.

3. After generation 1, the Bulmer effect will reduce the genetic variance, which indirectly reduces the accuracy of selection. The accuracy of selection increases when more information on the marker effects becomes available over generations, and this effect is larger in the selection scheme than in the random selection scheme, probably because the favourable alleles become more abundant and are thus more accurately estimated. Therefore, the accuracy of selection in generation 5 for genomic selection (0.823) is similar to the accuracy of selection for random selection (0.811) in generation 5 for the basic scheme.

4. Increasing Ntested from 3000 to 6000 increased accuracy of selection in generation 5 from 0.823 to 0.845. With a lower heritability of 0.1, the effect of increasing Ntested from 3000 to 6000 (not shown previously), increased the accuracy of selection in generation 5 from 0.742 to 0.763. Hence, the increase in accuracy of selection was similar.

5. The reduction in accuracy of selection with FIRST-GEN was somewhat larger for the lowest marker density than for the two other marker densities.

## Conclusion

The results using the current sib-breeding design of family-based aquaculture breeding schemes, which was not optimised in any way for genomic selection, show that genomic selection yields high genetic gain, accuracy of selection and very low rates of inbreeding, which makes it a promising selection scheme. Various strategies were evaluated to reduce the amount of sib-testing and genotyping, to reduce costs, but all resulted in loss of the accuracy of selection and thus of genetic gain. Genotyping costs may also remain high in a near future, and further research on strategies to reduce the number of fish to genotype is highly needed.

## Competing interests

The authors declare that they have no competing interests.

## Authors' contributions

AKS wrote the main computer program, ran computer programs and drafted the manuscript. THEM wrote computer modules for genome-wide breeding value estimation and for Fisher-Wright populations and helped to draft the manuscript. Both authors have approved the final manuscript.
